# ICS/Ultra LABA in the Treatment of Obstructive Airway Diseases: A Consensus of Indian Experts

**DOI:** 10.3390/arm90050051

**Published:** 2022-09-29

**Authors:** Raja Dhar, Deepak Talwar, Prince James, Ashwini Mishra, Judo Vachaparambil, Saiprasad Patil, Nishtha Khatri, Sagar Bhagat, Hanmant Barkate

**Affiliations:** 1 Department of Pulmonology, The Calcutta Medical Research Institute, Kolkata 700088, India; 2 Metro Respiratory Center, Pulmonology & Sleep Medicine, Metro Hospitals & Heart Institute, Noida 201301, India; 3 Interventional Pulmonology Department, Naruvi Hospital, Vellore 632004, India; 4 Department of Tuberculosis and Chest Medicine, Baba Raghav Das Medical College, Gorakhpur 273013, India; 5 Department of Pulmonary Medicine, Sun Medical Research Centre, Thrissur 680021, India; 6 Global Medical Affairs, Glenmark Pharmaceuticals Ltd., Mumbai 400099, India

**Keywords:** Asthma, COPD, consensus, ICS/ultra LABA, FF/Vi, vilanterol, fluticasone furoate

## Abstract

**Highlights:**

**What are the main findings?**

- FF/Vi is an effective choice for patients who are uncontrolled on their current Asthma treatment and also for controlled Asthma patients.
- SABA or ICS/SABA can be a viable reliever option when personalizing treatment for appropriate sets of patients with FF/Vi.

**What is the implication of the main finding?**

- FF/Vi offers a definitive advantage in terms of treatment adherence and compliance benefit as compared to the conventional ICS/LABAs.
- Adding LAMA to FF/Vi (100/25 μg) in GOLD group D and B COPD patients can be an optimized strategy.

**Abstract:**

Inhaled corticosteroid and ultra-long-acting beta-agonist (ICS/uLABA) combination is a recent advancement in the armamentarium against obstructive airways diseases (OADs). The combination of ICS/uLABA has several advantages, creating a favorable landscape for its utilization. Fluticasone furoate/vilanterol trifenatate (FF/Vi) is one such example of an ICS/uLABA. It offers several benefits from both drugs, such as a convenient once daily dosing schedule; high lipophilicity; high receptor affinity of fluticasone furoate along with high functional selectivity and a quick onset of action of vilanterol. However, the Global Initiative for Asthma (GINA) as well as the Global Initiative for Chronic Obstructive Lung Disease (GOLD) guidelines do not clearly define the positioning of ICS/uLABA compared to conventional ICS/LABAs. There are a few areas of uncertainty especially around the appropriate reliever strategy with ICS/uLABA in Asthma. The current consensus was planned with a group of Indian pulmonology experts to provide more clarity on the potential use of FF/Vi in Asthma and COPD. The clinical statements highlighted in this consensus manuscript address crucial clinical questions revolving around the efficacy and safety of FF/Vi as compared to conventional ICS/LABAs and identify the ideal patient profile for its use. This consensus paper also sheds light upon the appropriate reliever to be used along with FF/Vi in Asthma and the utilization of FF/Vi-based triple therapy in OADs. Expert recommendations mentioned in this paper will serve as guidance to pulmonologists as well as consultant physicians who are involved in providing care to OAD patients and will help them weigh the various factors that need to be taken into account while prescribing ICS/uLABA combination.

## 1. Background 

Obstructive airway diseases (OAD) are a leading cause of mortality, morbidity, and disability-adjusted life years (DALYs) worldwide. Chronic inflammation and obstruction of the airways are common in all types of OAD. As a result, inhalational corticosteroids and bronchodilators are the mainstay of therapy in the overall management of OAD. Asthma and chronic obstructive pulmonary disease (COPD) are chronic diseases and necessitate daily maintenance medications for management of symptoms and better quality of life. Despite decades-long experience with the current management for Asthma and COPD, treating physicians still face challenges in terms of adherence, compliance, and complete control of symptoms.

Medication non-adherence rates among patients with Asthma and COPD are as high as 30 to 70 percent [1,2]. More than once-daily dosing results in lower adherence rates than medications dosed once daily [1]. Until recently, all commercially available inhaled corticosteroid and long-acting beta-agonist (ICS/LABA) fixed-dose combination (FDC) preparations in India were intended for twice- or three-times daily use. With introduction of the first ICS/ultra LABA (ICS/uLABA) FDC in the form of Fluticasone furoate/Vilanterol (FF/Vi), physicians now have the choice of prescribing once-daily ICS/uLABA for their patients. 

Vilanterol (Vi), being an ultra LABA, has unique pharmacological properties as compared to other molecules of the same class, as shown in Table 1 [3,4]. Vi is a highly lipophilic β2-agonist with inherent 24-hour activity in vitro, which is in development for once-daily administration in combination with fluticasone furoate (FF) for both Asthma and COPD. Vi shows a level of intrinsic efficacy that is significantly greater than that of salmeterol but comparable to indacaterol. Intrinsic efficacy is the extent to which LABAs activate the receptor without regard for drug concentration or receptor numbers. In isolated human small airways, Vi has a significantly faster onset of action and a significantly longer duration of action than salmeterol, with a significant bronchodilator effect observed even at 22 h [5].

Fluticasone furoate has high lipophilicity, high tissue permeability, low solubility and slow inhaled-drug-particle dissolution. It has high affinity for glucocorticoid receptor (GR) and dissociates slowly from it. Only around half the quantity of FF is needed to occupy the same number of glucocorticoid receptors compared to fluticasone propionate (FP). This allows for greater potency with reduced drug exposure [6,7]. FF exhibits greater in-vitro anti-inflammatory potency. It is a stronger inhibitor of NF-κB activation and TNF release than mometasone furoate, budesonide and ciclesonide’s active metabolite, and has a longer duration of action than FP in inhibiting activation of NF-κB and AP-1 [5].

The fluticasone furoate/vilanterol (FF/Vi) (100/25 µg) dry powder inhaler (DPI) has already been approved as maintenance therapy for both COPD and Asthma by the EMA and the US FDA, while the FF/Vi (200/25 µg) DPI has been approved for Asthma management [8,9]. FF/Vi DPI is the first once-daily ICS/uLABA recently commercialized in India for the treatment of COPD (FF/Vi: 100/25 µg) and Asthma (FF/Vi: 100/25 µg and 200/25 µg) [10]. Landmark studies for FF/Vi in COPD and Asthma found improved symptom control, improved adherence, and a lower need for rescue medication with FF/Vi than with conventional ICS/LABA, including budesonide/formoterol (BUD/FOR) [11,12,13,14]. 

Global Initiative for Asthma (GINA) guidelines have now shared BUD/FOR as the preferred therapeutic option throughout the management steps in asthmatic patients, but a thorough discussion of the important role of ICS/uLABA in Asthma management is lacking. Similarly, the Global Initiative for Chronic Obstructive Lung Disease (GOLD) guidelines do not highlight the benefit of ICS/uLABA vis-a-vis conventional ICS/LABA [15,16]. The current consensus was conducted by Indian experts with an intent to unlock the full potential of the novel combination of ICS/uLABA in the management of Asthma and COPD. 

## 2. Methodology

A roundtable meeting of experts was held to formulate recommendations for the present role of inhaled FF/Vi (ICS/uLABA) treatment in patients with Asthma and COPD. This expert panel consisted of 20 pulmonologists. The panel was invited based on their clinical experience, academic achievements and engagement in clinical research in the area of OAD management. A minimum of ten years of clinical experience in the field was a mandatory requirement.

The expert panel was tasked with examining the reviewed literature around FF/Vi in Asthma and COPD, identifying areas of ambiguity in its use to develop clinical consensus statements and formulating practice recommendations through a roundtable discussion.

After discussion with the faculty members, the following clinical consensus statements were formulated. The statements are depicted in Table 2.

To develop clinical consensus statements, an electronic search of the PubMed and Embase database was undertaken around each scientifically important topic. A thorough literature search was carried out to discover relevant English-language publications published between 1 January 2010 and 31 August 2021.Various combinations of keywords such as “fluticasone furoate/vilanterol,” “FF/Vi,” “ICS/ultra LABA” and “ultra LABA/ICS” were used. Appropriate variations in search phrases “fluticasone furoate”, “vilanterol,” and Boolean operators (AND, OR) were used wherever possible.

The findings of the literature searches, including electronic full-text copies, were sent to the panel members. A roundtable meeting was held in September 2021 and the consensus statements were deliberated upon by the moderator. Expert opinion was sought from the panel in the form of agreement (defined as more than 70% panelists agreeing), conditional agreement (defined as 50–70% panelists agreeing) or disagreement (defined as more than 50% panelists disagreeing). The meeting included presentation of the evidence and a debate.

**Figure 1:**
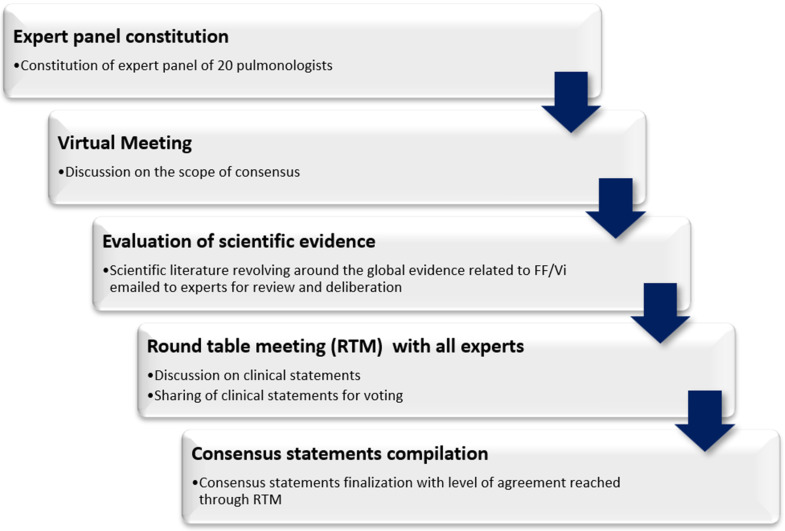
Stepwise consensus development process.

Finally, the consensus report was written, peer reviewed (see Acknowledgements), and comments were included as the authors felt suitable. Figure 1 provides an overview of the consensus development process.

The class of recommendation and level of evidence grading used in this manuscript are based on the grading system used by Knuuti et al. which was modified for suitability in the current study [17]. The same has been depicted in Table 3 below. 

## 3. Results and Discussion

The combination of FF/Vi is approved by various leading regulatory authorities in two strengths (100/25 μg and 200/25 μg) as a dry powder inhaler (DPI) formulation. The global approval status and indication of FF/Vi combinations is depicted in Table 4. 

Table 5 depicts the summary of critically analyzed literature pertaining to FF/Vi including randomized clinical trials, systematic reviews, meta-analysis and observational studies.

Altogether, there were eight clinical consensus statements (four for Asthma and four for COPD). A key summary of recommendations are discussed below.

### 3.1. Asthma

#### 3.1.1. Comparison of FF/Vi to Current Gold Standard (BUD/FOR) in Asthma 

The current Global Initiative for Asthma (GINA) strategy document recommends using ICS/Formoterol combination both as a daily controller and as a rescue medication (MART) as a preferred strategy. Recommendations by GINA represent a global strategy document but not a guideline; therefore, there is a need for a treatment strategy based on professional judgment, population characteristics and local healthcare systems.

MART regimen rely on patient perceptions to control symptoms. It can be unreliable, as the underlying inflammation may be present despite the absence of symptoms. MART requires a considerable degree of patient education to explain the role of treating inflammation and smooth muscle constriction with the use of a single inhaler [24,25]. 

Moreover, use of MART therapy does not rule out the use of SABA completely. The multinational APPaRENT survey conducted in patients and physicians showed that 55–75% of physicians reported awareness towards MART dosing. Yet, the majority of them reported that they prescribed a SABA along with MART at some point in time. As far as the patients are concerned, only 20–50% of the patients were aware about the appropriate MART dosing strategy [26].

There are some limitations of the MART strategy. Patients have to play a more active role in their disease management. Identification of eligible patients for the MART strategy in clinical practice is complicated, especially when a patient moves up the treatment ladder (step 3–5). Adjustment of treatment is challenging, with no clarity on when and how much to step up or down. In fact, fewer than 1 out of 5 patients remain well controlled at 1 year with BUD/FOR MART therapy, suggesting the underlying issue remains unresolved [24,27]

Therefore, clinicians should assess and personalize treatment plans to ensure adherence and appropriate treatment of the modifiable risk factors.

FF/Vi versus BUD/FOR and conventional ICS/LABA

A total of 25% more patients experienced an improvement in their Asthma control with FF/Vi versus conventional ICS/LABAs, including BUD/FOR in the Salford Lung Study (SLS) [22].

Asthma control is not just about reducing symptoms:

- FF/Vi can help Asthma patients reduce their rate of exacerbations versus both BUD/FOR and ICS, as seen in real-world and clinical settings. FF/Vi has 13% lower risk of overall exacerbation versus BUD/FOR (*p* < 0.001) and 22% lower risk of severe exacerbation versus BUD/FOR (*p* = 0.027) [14,28].
- Rescue-medication use is a key indicator of Asthma control. When compared with BUD/FOR, FF/Vi was associated with a 10% reduction in the use of rescue medication (SABA) in real-world settings [14].
- When measured using the Asthma Quality of Life Questionnaire (AQLQ), 27% more patients improved their quality of life versus conventional ICS/LABAs in everyday practice [29].

An estimated 71% of patients with Asthma remain uncontrolled despite treatment [19]. The problem lies with many Asthma patients over-perceiving their level of Asthma control when, upon deeper discussion, their symptoms often turn out to be frequent and regular [30].

A high proportion of patients who are thought to have “difficult” or therapy-resistant Asthma are found to have poor adherence to maintenance therapies when investigated. Such individuals are, thus, difficult asthmatics rather than treatment resistant. Non-adherence with such therapies may result in reduced lung function, increased-interval symptoms, and more frequent/severe Asthma attacks [31]. Expert panel recommendations on the use of FF/Vi in asthma are depicted in Box 1.

Box 1 **Recommendation.**

**Recommendation**

FF/Vi has shown improved treatment control and lesser SABA dependence compared to other ICS/LABAs including BUD/FOR.

1. For uncontrolled asthma patients (Step 3-4) on BUD/FOR → Switch to FF/Vi (IA)
2. Stable Asthma (Step 3–4) on BUD/FOR → Shared decision making with patient on choice of treatment (IIC)

FF/Vi isn’t just an effective choice for patients who are uncontrolled on their current asthma treatment—it can be a considerate choice for controlled asthma patients too.

#### 3.1.2. Reliever Use with FF/Vi in Asthma Patients

An anti-inflammatory and reliever (AIR) approach (BUD/FOR) has been shown to be more effective than SABA-ICS or LABA-ICS plus SABA in reducing risks of severe Asthma exacerbation and providing similar levels of day-to-day Asthma control; however, there is no such evidence vis-a-vis ICS/uLABA to our knowledge.

Limitations of the AIR strategy include difficult identification of eligible patients in clinical practice, especially when a patient moves up the treatment ladder (step 3–5) and challenging adjustment of treatment with no clarity on when and how much to step up or down [27].

The relative airway potency of FF was found to be greater than predicted from its relative glucocorticoid receptor (GR) binding affinity compared to FP and BUD. This may result in enhanced airway potency, prolonged reduction in airway sensitivity and a reduced need for reliever use [14]. 

Once-daily dosing with FF/Vi has shown to provide continuous 24-hour efficacy with no drop in effectiveness towards the end of the 24-hour dosing interval [32,33,34]. Hence, the requirement of a reliever SABA when using FF/Vi would be minimal, if existing at all.

Expert panel recommendations on the appropriate reliever use with FF/Vi are depicted in Box 2.

Box 2 **Recommendation.**

**Recommendation**

Most experts supported the use of SABA as a reliever option when personalizing treatment with FF/Vi for appropriate set of Asthma patients.

1. For treatment naïve patients, SABA must be used as a reliever with FF/Vi (IA)
2. For previously BUD/FOR treated patients switched to FF/Vi as maintenance, ICS-SABA or BUD/FOR to be used as reliever (IIC)

#### 3.1.3. Adding OD LAMA to FF/Vi or High Dose FF/Vi in Uncontrolled Severe Asthma as Optimized Strategy

The addition of LAMA to FF/Vi (100/25) resulted in improved lung function and symptom control in moderate or severe uncontrolled Asthma [23,35].

- Adding LAMA to FF/Vi improved lung function in patients with moderate or severe Asthma uncontrolled on conventional ICS/LABA. There are higher odds of Asthma control when LAMA is added to FF/Vi. The mean annualized rate of exacerbations was found to be 0.31 for both FF/Vi/UMEC (100 and 200) and for FF/Vi (100 and 200), with a 2.6% reduction in rate with FF/Vi/UMEC compared with FF/Vi.
- ACQ-7 responders at 24 weeks were numerically higher (62%) with LAMA added to FF/Vi (100/25) as compared to higher dose of FF/Vi (200/25) when used alone (58%)
- Numerically greater improvements in clinic-trough FEV1 were observed with FF/UMEC/Vi 100/62.5/25 μg versus FF/Vi 200/25 μg across the baseline eosinophil and FeNO ranges.
- There is now substantial evidence on the efficacy and safety of LAMAs in uncontrolled Asthma notwithstanding treatment with ICS/LABA combinations. This regimen is recommended by GINA as an optimization step for patients with severe Asthma before any biologic or systemic corticosteroid treatment is initiated.

Box 3 represents the expert panel recommendations on the addition of LAMA to FF/Vi. Recommendations on stepping up to a higher dose of FF/Vi are also shown in Box 3.

Box 3 **Recommendation.**

**Recommendation**

1. Addition of LAMA to FF/Vi should be considered preferentially for patients with persistent airflow limitation and bronchodilator reversibility, independent of blood eosinophil and/or FENO levels (IIA)
2. Step-up to high-dose ICS should be considered particularly in patients with increased eosinophil (>300 cells/μL) and/or FENO levels → FF/Vi (100/25) to FF/Vi (200/25) (IA)

#### 3.1.4. Triple Therapy in Asthma—Open-Triple Versus Single-Inhaler-Triple Therapy?

**Figure 2:**
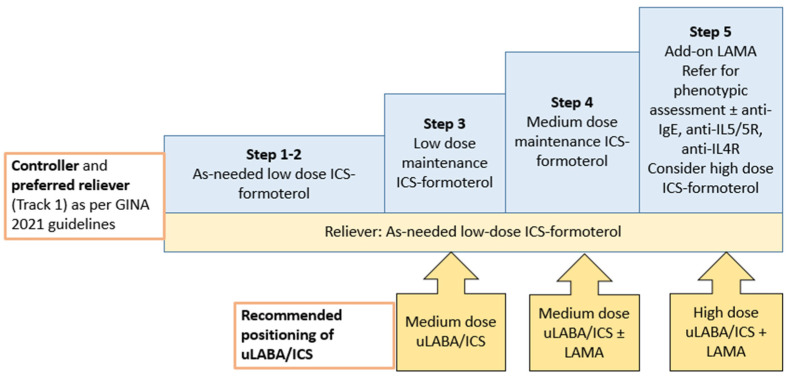
Various steps of GINA 2021 guidelines where use of uLABA/ICS can be considered.

ICS/LABA/LAMA FDCs are effective and safe in uncontrolled Asthma, and the dose of ICS in the combination portrays itself as the discriminating factor while treating patients with a history of moderate or severe exacerbation. This has been depicted in Figure 2.

A recent network meta-analysis showed that triple-combination therapies with an ICS administered at high dose (HD) were superior (*p* < 0.05) to MD ICS/LABA/LAMA FDC in preventing severe exacerbation (relative risk 0.46–0.65), but not with regards to moderate exacerbation (p>0.05).

These findings suggest that triple combinations including an ICS when given at an HD may represent the first treatment choice in patients with a history of severe Asthma exacerbation, whereas in patients with a history of moderate Asthma exacerbation, either MD ICS/LABA/LAMA FDC or HD ICS/LABA FDC may be used as a first-line treatment.

Despite the unpredictable nature of Asthma exacerbations regardless of disease severity, recent evidence indicates that the past history and severity of exacerbations in the year prior may predict the risk and severity of future Asthma exacerbations. 

In this respect, the results of the subset analysis on exacerbation severity may provide the rationale for a tailored therapy based on the severity of exacerbation that each patient experienced in the previous year, thus leading to the optimization of the dose of ICS and the number of bronchodilators included in the FDC [36]. Box 4 depicts the expert panel recommendations on the use of triple therapy in asthma.

Box 4 **Recommendation.**

**Recommendation**

1. Poorly controlled symptomatic patients could be effectively treated with triple combination therapies including different strengths of an ICS according to the severity of previous exacerbations (IA)
2. Physicians may exercise both open triple and SITT options in clinical practice as per the clinical need and patient preference (IC)

Open triple therapy advantages in clinical practice

- Flexibility for dose titration, especially of ICS
- Flexibility for choice of components
- Split ICS/LABA & LAMA as am/pm dosing in select patients

Single inhaler triple therapy advantages in clinical practice

- Optimal synergy benefit of giving LABA/LAMA/ICS together
- Better compliance due to less frequent dosing
- Reduced device related errors

### 3.2. COPD

#### 3.2.1. Profiling of the Patients for Use of FF/Vi in COPD

Initiating treatment for COPD, similar to Asthma, with the combination of a bronchodilator and an ICS is very common in primary-care settings.

Though ICS use in COPD is limited on account of the inherent differences in the inflammatory process involved in COPD and Asthma, certain patient profiles have superior clinical benefit as oppose to avoiding it. ICS can be added to LABA or to the combination of LABA and LAMA leading to TT. GOLD guidelines strongly suggest the use of ICS in patients with—a history of hospitalization(s) for exacerbation of COPD, ≥ two moderate exacerbations per year, baseline eosinophil count (BEC) ≥300 cells/µL and history of or concomitant Asthma. GOLD also advise to consider the use of ICS in patients with—history of one moderate exacerbation per year, BEC ≥ 100–< 300 cells/µL. For patients with COPD, in particular those who continue to smoke, the bronchial epithelium and infiltrating immune cell types may be steroid-insensitive, rendering ICS-containing therapies ineffective [37].

In SLS-COPD study with predominant group B, D patients, FF/Vi has shown benefits over and above conventional ICS/LABA. Rates of moderate or severe exacerbation reduced significantly (*p* = 0.02) with FF/Vi compared to UC. Those patients who were switched from an ICS-containing regimen (mostly FP/SAL or BUD/FOR) showed the greatest exacerbation benefit. An improvement in COPD-related health status (i.e., ≥ two reduction in CAT score) was experienced by 45% in the FF/Vi group versus 36% in the UC group (OR 1.51, P<0.001). This was further confirmed by a large observational study.

Expert panel recommendations on the appropriate patient profiles for use of FF/Vi in COPD have been depicted in Box 5.

Box 5 **Recommendation.**

**Panel recommendations**

**Figure 3:**
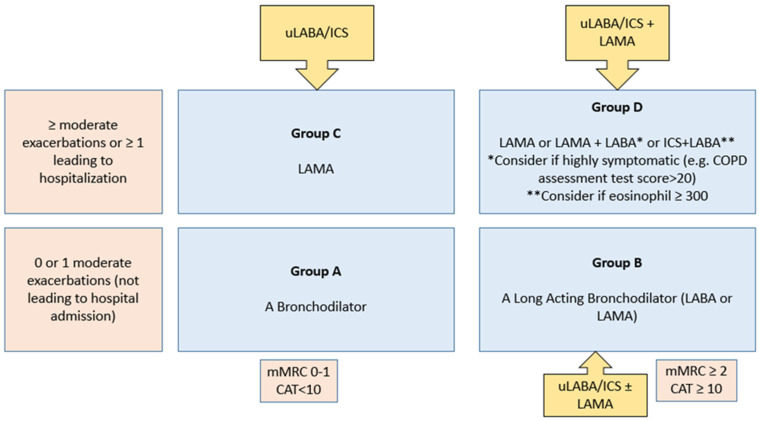
Potential use of FF/Vi in the various GOLD 2021 categories of COPD patients. mMRC: Modified Medical Research Council Dyspnea Scale; CAT: COPD Assessment Test.

1. ICS use based on clinical and biomarker profiling of COPD patient. FF/Vi has potential to use across clinical spectrum of COPD proposed by experts is as below and in Figure 3.
  - GOLD Group D—FF/Vi can be used with add on LAMA (IA)
  - GOLD Group B—FF/Vi can be used without or with add on LAMA driven by BEC, smoking status, ACO, pre-existing ICS therapy (IIA)
  - GOLD Group C—FF/Vi alone can be used (IIB)
  - Pre-existing ICS/LABA therapy—Switch to FF/Vi after patient profiling by shared decision making (IIC)

#### 3.2.2. Pneumonia Risk with FF/Vi in COPD

Acute exacerbation of COPD (ECOPD) and pneumonia often present with similar symptoms. This poses a diagnostic challenge and can significantly impact on patient outcomes. At times, pneumonia and ECOPD can exist simultaneously, making it difficult to understand which superseded the other. 

The modelled probabilities of an ECOPD over a 1-year period were considerably higher than those for pneumonia. The seemingly paradoxical observation that ICS reduce exacerbation frequency but increase pneumonia risk suggests that ECOPD and pneumonia may have different underlying aetiologies in individual patients [38,39].

A pooled analysis from five clinical studies showed that the probability of ECOPD was considerably higher than for pneumonia. The above factors must be taken into consideration while assessing treatment-associated pneumonia-risk-exacerbation benefit assessment with ICS [38]. The European Medicines Agency states that there is no conclusive clinical evidence for intra-class differences in the magnitude of the risk among ICS products [40]. Whether one should give due importance to the risk of pneumonia with an individual ICS still remains questionable. 

Evidence suggests that ICS may increase the risk of pneumonia and that the same is mainly linked to fluticasone propionate use in moderate to severe COPD patients, as seen in the TORCH study. Nevertheless, an increased risk of pneumonia with ICSs was not seen in more recent large studies conducted in moderate than in severe patients [41].

- A real-world effectiveness study in COPD patients showed that there was no increase in the risk of pneumonia in FF/Vi group as compared to the usual care group [18].
- Another large clinical trial conducted in patients with moderate COPD and heightened cardiovascular disease risk showed that the incidence of pneumonia was comparable in the placebo, fluticasone furoate, vilanterol and FF/Vi groups [20].
- A recent study showed that the risk of pneumonia with fluticasone furoate 100 μg (RR = 1.39) was significantly lesser than the 200 μg dose (RR = 1.90). The same study also showed that fluticasone furoate 100 μg was safer in terms of pneumonia risk as compared to fluticasone propionate 500 μg (RR = 1.80) and 1000 μg (RR = 1.64) and was closer to 800 μg budesonide (RR = 1.26) [41].

Box 6 represents the expert panel recommendations on risk of pneumonia with FF/Vi.

Box 6 **Recommendation.**

**Panel recommendations**

1. There is a clinical dilemma while diagnosing exacerbation vs pneumonia in COPD patients. Along with clinical signs, radiological diagnosis must be considered for distinctive diagnosis of pneumonia while ruling out exacerbation (IA)
2. The choice of the ICS prescribed should not solely rely upon background incidence of pneumonia in COPD patients. An individualized risk benefit assessment should be performed considering risk of exacerbation (IA)
3. There is a lesser risk of pneumonia with fluticasone furoate (100 µg) as compared to fluticasone propionate in COPD patients (IA)

However, the panel also mentioned that the clinical significance of the varying incidence of pneumonia with different ICS needs to be explored further

#### 3.2.3. Benefits of FF/Vi (ICS/uLABA) as Compared to Conventional ICS/LABA Combinations

(a) Exacerbation reduction

Frequent exacerbations in patients with COPD are a major reason for disease progression and mortality [42]. Evidence suggests that patients who had experienced a greater exacerbation burden after initiation of maintenance therapy had a worse lung function at diagnosis as well as a more rapid lung function decline. This emphasizes the need for better treatment options and treatment strategies [43].

- Real-world evidence suggests that FF/Vi provides a significant exacerbation benefit. A large-scale effectiveness study showed that the rate of moderate or severe exacerbations is significantly lower, by 8.4% in the FF/Vi group as compared to the usual care group. Patients switched from an ICS-containing regimen (FP/SAL or BUD/FOR) showed the greater benefit [18].
- Further evidence in the form of a clinical trial showed that FF/Vi with or without LAMA had better and consistent protection than LABA/LAMA group against moderate-to-severe exacerbations in patients with increasing baseline eosinophil count [19].
- A large observational study showed that FF/Vi was associated with a 14% lower risk of having a COPD-related moderate or severe exacerbation compared to BUD/FOR [21].
- As far as the single inhaler triple combination is concerned, rate ratio for moderate to severe exacerbations in FF/VI/UMEC group was comparable to that of BUD/FORM/Glycopyrronium (rate ratio = 0.99 in both the groups) [44].

(b) Lung-function improvement

COPD is a progressive inflammatory disease causing a gradual decline in expiratory flow and, thereby, a deterioration in lung function [45]. It is associated with a significant and rapid deterioration of lung function and a great degree of airway obstruction [46].

The role of FF/Vi in lung function slowing lung function decline is well-established.

- A clinical trial in COPD patients showed that the rate of lung-function decline decreased by 8 mL/year in the FF/Vi group as compared to the placebo group (*p* = 0.019) [20].
- Evidence also shows that weighted mean (wm) FEV1 (mean 130 mL) is greater and time to 100 mL improvement shorter (median 16 min) with FF/Vi as compared to FP/SAL (weighted mean 108 mL, median 28 min) [47].
- A recent study showed that once-daily FF/Vi improved trough FEV1 by about 230 mL as compared to a placebo in COPD patients. However, other trials suggest a more modest increase (100–130 mL) in FEV1 [48].

(c) Symptom control

COPD assessment tool (CAT) is often used to gauge the symptom prognosis and health status recovery in COPD patients [49]. FF/Vi has shown beneficial effects in this aspect. A real-world phase III study showed an improvement in COPD-related health status (a decrease in CAT score of more than or equal to 2): 45% in FF/Vi group versus 36% in UC group (OR 1.51, *p* < 0.001) [18].

(d) Quality-of-life improvement

COPD impairs quality of life and day-to-day functioning of patients. Improvement in the quality of life of COPD patients is an important goal and the same has been emphasized in GOLD guidelines [16]. A systematic review showed that with FF/Vi 100/25 μg, FP/SAL 500/50 μg and BUD/FORM 400/12 μg, all three groups showed significant improvement in mean SGRQ score as compared to a placebo. The mean improvement with FF/Vi (−4.6 units) exceeded the minimal clinically important difference (MCID) of 4 units and was numerically higher than that seen with FP/SAL (−3.278) or BUD/FORM (−3.64) in COPD patients [50].

Mortality benefit

- IMPACT trial was the first large trial reporting mortality data with the use of triple therapy in the treatment of COPD. The trial showed that triple therapy offered significant mortality benefits as compared to LABA/LAMA (HR = 0.58). The trial also showed that dual therapy with FF/Vi provided a significant mortality benefit versus LABA/LAMA (HR = 0.61) [51].
- The SUMMIT trial was conducted in patients with moderate COPD and heightened cardiovascular disease risk showed that all-cause mortality with FF/Vi was 12.2% lower as compared to a placebo group but was not statistically significant (*p* = 0.137). However, the all-cause mortality was significantly lower in the FF/Vi group (HR = 0.76) among subjects with a Summit Score of 14 to 19. The SUMMIT trial is one of the largest randomized controlled COPD trials, with more than 16,000 patients [20].
- The results of recent network meta-analysis provides high quality evidence that both MD ICS/LABA/LAMA and MD ICS/LABA FDCs were significantly effective in reducing on-treatment all-cause of death, whereas only MD ICS/LABA/LAMA FDC significantly prevented adjudicated cardiovascular mortality. Indeed, the protective effect against mortality was related to the dose of the ICS in the FDC. A total of ~125 COPD patients had to be treated for one year with an MD ICS-containing combination to prevent one death compared with LABA/LAMA FDC. Given the importance of the outcome “mortality”, the specific context of COPD characterized by a high prevalence, and the safety profile of MD ICS-containing FDCs, an NNT of 125 seems to be more than acceptable and of clinically relevant magnitude [52].

(e) Safety benefits

Cardiovascular events are regarded as an important source of morbidity and mortality in COPD patients, regardless of the disease severity. COPD is, in fact, considered a powerful, independent risk factor for cardiovascular morbidity and mortality. Evidence indicates that cardiovascular diseases may account for 25% of all deaths and around 40–50% of all hospitalizations in patients with mild to moderate COPD. Therefore, for therapies positioned to reduce all-cause mortality and hospitalizations in COPD, it is desirable that they have beneficial effects on the cardiovascular system [53]. A pilot-randomized controlled trial concluded that the short-term use of FF/Vi led to consistent and physiologically plausible beneficial effects on cardiac structure, function, and pulmonary vasculature [54].

(f) Adherence and compliance benefit

A decline in adherence to COPD treatment appears early right from the first year of treatment, with a steep decrease from the beginning, which continues afterwards. Hence, it would be prudent to intervene early to maximize adherence and subsequent benefit from the therapy [55]. Patients as well as physicians play a major role in ensuring a good adherence. Physicians can affect treatment adherence in COPD based on the medication class prescribed, method of administration, dosing regimen and polypharmacy [56]. Use of once daily FF/Vi regimen is one such early implementable intervention directed towards improving the dosing regimen taking into account that the BUD/FOR combination has a twice-daily dosing schedule. Along with a favorable safety and efficacy profile, the added convenience of once-daily administration schedule of FF/Vi will prove to be beneficial and improve adherence in COPD patients [48]. Box 7 shows expert panel recommendations on the advantages of FF/Vi.

Box 7 **Recommendation.**

**Panel recommendations**

1. The exacerbation and symptom control benefit showed by FF/Vi in the real world studies outweighs the benefit from usual ICS/LABAs (IIA)
2. FF/Vi to be used over usual ICS/LABAs in patients with cardiovascular morbidity because of its beneficial effects on the cardiovascular system (IB)
3. FF/Vi offers a definitive advantage in terms of adherence and compliance benefit as compared to the other ICS/LABAs (IA)
4. Since COPD is a chronic disease and needs long term treatment, experts suggested that once daily dosing would be the greatest advantage offered by FF/Vi (IC)

#### 3.2.4. Triple Therapy in COPD—Open-Triple Versus Single-Inhaler-Triple Therapy?

There is evidence to validate that triple therapy with FF/Vi/UMEC results in a lower rate of moderate or severe COPD exacerbations than ICS/LABA or LABA/LAMA in symptomatic COPD patients with a previous history of exacerbation [57]. A randomized non-inferiority study in COPD patients showed that single-inhaler triple therapy (FF/Vi/UMEC) was non-inferior to ICS/LABA or LABA/LAMA inhalers with respect to trough FEV1 change from baseline at 24 weeks. Similar results were seen on all other measures pertaining to efficacy, quality of life and safety [58]. However, the phenotype of patients who will require an add-on LAMA to FF/Vi in clinical practice is not clearly established. 

Open-triple therapy is primarily useful in the patients who prefer flexibility in dose titration and thereby show a preference towards splitting the components. However, for patients predisposed towards poor adherence, single-inhaler triple therapy could be considered. A recent study showed that bronchodilation and lung-function improvement was comparable in the single-inhaler triple therapy and open triple therapy [59]. Box 8 represents expert panel recommendations on the use of triple therapy in COPD.

Box 8 **Recommendation.**

**Panel recommendations**

1. Add LAMA to FF/VI (100/25 μg) in group D and B COPD patients as an optimized strategy (IIA)
  However, the de-escalation strategy needs to be defined while considering an improvement in clinical symptoms.
2. Open triple therapy to be a good strategy in unstable COPD (post exacerbation requiring hospitalization) patients as it allows to titrate the dose of ICS, it also gives flexibility to prescribe ICS/LABA in the morning and LAMA in the evening in those where exacerbation is predominant over dyspnea (IC)

The benefits of both open triple and SITT agreed by experts are same as discussed under asthma section.

Challenges and perspectives of ICS/uLABA approach [60].

Along with Fluticasone furoate/Vilanterol, Indacaterol/Mometasone combination is another example of ICS/uLABA combination which has expanded the armamentarium of OAD treatment. A convenient dosing schedule and compliance benefits are the clear advantages of the ICS/uLABA combinations. Moreover, ICS/uLABAs appear to be better treatment options compared to conventional ICS/LABAs based on several clinical studies in Asthma as well as in COPD. Yet, there are certain challenges associated with the use of ICS/uLABAs in practice.

(a) International guidelines (GINA/GOLD) do not lay much importance and do not differentiate ICS/uLABAs from conventional ICS/LABAs.
(b) Clinical studies comparing SMART versus ICS/uLABAs in Asthma patients are lacking.
(c) Possibility of using FF/Vi as a SMART strategy considering its quicker onset of action needs to be evaluated.

## 4. Conclusions

Recent evidence indicates that ICS/uLABA have laid the foundation for an optimistic change in the current landscape of OAD management. The current consensus is a sincere effort to understand the evolving role of ICS/uLABA in treatment of OADs. This consensus statement developed from a composite approach of the expert panel will provide a guiding light to pulmonologists and respiratory physicians and will help them weigh the various factors that need to be taken into account while prescribing ICS/uLABA. Experts believe that the recommendations provided here can be considered in the OAD management guidelines. We also indicate the need for the implementation of programs to sensitize pulmonologists and respiratory physicians towards the effective implementation of the recommendations for the utilization of ICS/uLABA. The landscape of ICS/uLABA can be further optimized with clinical experience.

## Figures and Tables

**Table 1:** Comparison of distinguishable pharmacological properties of LABAs and uLABAs.

LABA/uLABA	Functional Selectivity (β2:β1)	Onset of Action (Minutes)
Formoterol	130	5.9
Salmeterol	1595	13.7
Indacaterol	12.5	10.9
Vilanterol	2400	3.45 min
Summary: Vilanterol has a greater functional selectivity and quicker onset of action as compared to other LABAs/uLABA		

**Table 2:** Clinical statements formulated for expert recommendations.

**Asthma**
Comparison of FF/Vi to current Gold Standard (BUD/FOR) in Asthma Reliever use with FF/Vi in Asthma patients Addition of once daily (OD) long-acting anti-muscarinic agent (LAMA) to FF/Vi or high-dose (HD) FF/Vi in uncontrolled severe Asthma as optimized strategy Triple therapy in Asthma—open-triple versus single-inhaler-triple therapy
**COPD**
Profiling of the patients for use of FF/Vi in COPD Pneumonia risk with FF/Vi in COPD Benefits of FF/Vi (ICS/uLABA) as compared to conventional ICS/LABA combinations Triple therapy in COPD—open-triple versus single-inhaler-triple therapy

**Table 3:** Class of recommendation and level of evidence.

**Class of Recommendation**		**Consensus Response**
I	Evidence and/or general agreement that a given treatment or procedure is beneficial, useful, effective	Agreement (It is recommended or is indicated)
II	Conflicting evidence and/or a divergence of opinion about the usefulness/efficacy of the given treatment or procedure	Conditional agreement (May be considered)
III	Evidence or general agreement that the given treatment or procedure is not useful/effective, and in some cases may be harmful	Disagreement (It is not recommended)
**Level of evidence**		
A	Data derived from multiple randomized clinical trials or meta-analysis	
B	Data derived from a single randomized clinical trial or large non-randomized studies	
C	Consensus of opinion of the experts and/or small studies, retrospective studies, registries	

**Table 4:** List of FF/Vi combinations approved globally.

	Indication (Year of Approval)	
Regulator	FF/Vi (100/25 μg)	FF/Vi (200/25 μg)
US FDA	Asthma (≥18 years) (2015) and COPD (2013)	Asthma (≥18 years); 2015
EMA	Asthma (≥12 years) and COPD; 2013	Asthma (≥12 years); 2013
TGA	Asthma (≥12 years) and COPD; 2014	Asthma (≥12 years); 2014
PMDA	Asthma (≥12 years) (2013) and COPD (2016)	Asthma (≥12 years); 2013
DCGI, India	Asthma (≥12 years) (2017) * and COPD (2022)	Asthma (≥12 years); 2022
Summary: FF/Vi has been recently approved and marketed for Asthma and COPD in India, while it has been marketed in countries such as US, Europe, Australia, Japan since 2013.		

* FF/Vi first marketed in India since 2021.

**Table 5:** Summary of landmark clinical studies of FF/Vi in OAD.

Clinical Trial	Trial Design and Endpoints—Primary/Secondary	Patient Population	Treatment and Duration	Key Findings
**COPD**				
SLS COPD [18]	Phase 3, open-label, randomized, parallel-group effectiveness study Primary endpoint: Rate of moderate or severe exacerbations at 12 months among patients who had an exacerbation within 1 year before the trial Secondary endpoints (during the 12 months): ○ Rates of primary care contact and secondary care contact ○ Rates of treatment modification ○ Rate of exacerbations among patients who had an exacerbation within 3 years before the trial	Age ≥ 40 years Documented diagnosis of COPD One or more COPD exacerbations in the previous 3 years Those taking regular maintenance inhaler therapy, defined as the use of one or more long-acting bronchodilators	FF/Vi (100/25 μg) DPI (n = 1291) versus Usual-care (UC) group (n = 1309)	Rate of moderate or severe exacerbations was significantly lower by 8.4% with FF/Vi than UC Number needed to treat (NNT) = one additional moderate/severe exacerbation is prevented for every seven patients treated over 12 months Patients that had been switched from an ICS-containing regimen (mostly SF or FB) showed the greatest exacerbation benefit Improvement in COPD-related health status (≥2 ↓ed CAT score) 45% in FF/Vi group versus 36% in UC group (OR 1.51, *p* < 0.001) Severe exacerbations: no significant difference (*p* = 0.52) Time to first moderate–severe exacerbation: No significant difference (HR 0.93) No significant difference in the annual rate of COPD-related contact with primary care or secondary care contacts
IMPACT [19]	Phase 3, randomized, double-blind, parallel-group, multicenter trial Primary endpoint: annual rate of moderate or severe exacerbations during treatment ○ Secondary endpoints: change in trough FEV1 ○ Change in the St. George’s Respiratory Questionnaire (SGRQ) total score	Age ≥ 40 years Symptomatic COPD (COPD Assessment Test (CAT) score, ≥10 FEV1 < 50% of the predicted normal value and a history of at least one moderate or severe exacerbation in the previous year, or an FEV1 of 50–80% of the predicted normal value At least two moderate exacerbations or one severe exacerbation in the previous year.	Triple therapy fluticasone furoate-vilanterol-umeclidinium (FF/Vi/UMEC); n = 4151 versus umeclidinium–vilanterol (UMEC/Vi); n = 2070 versus FF/Vi; n = 4134)	The rate of moderate or severe exacerbations during treatment among patients assigned to triple therapy was 0.91 per year, as compared with 1.07 in FF/Vi (*p* < 0.001) and 1.21 per year in UMEC/Vi (*p* < 0.001). Among patients with eosinophil levels ≥ 150 cells/µL the exacerbation rate was 0.95 with triple therapy, 1.08 with FF/Vi, and 1.39 with UMEC/Vi. Difference between the triple-therapy and FF/Vi group was 97 mL while the difference between the triple-therapy and UMEC/Vi groups was 54 mL. Triple therapy group showed a response as defined by a decrease in the SGRQ total score of at least 4 points.
SUMMIT [20]	Phase III, prospective double-blind parallel group placebo controlled event-driven randomized trial Primary endpoint: all-cause mortality, secondary endpoints: on-treatment rate of decline in FEV1 and a composite of cardiovascular events consisting of on treatment cardiovascular death, myocardial infarction, unstable angina, stroke and transient ischemic attack.	Age: 40–80 years Smokers with at least a 10-pack-a-year history Post bronchodilator FEV1 between 50% and 70% FEV1/FVC ratio ≤ 0.7 mMRC score ≥ 2	FF/Vi (n = 4121) versus FF (n = 4135) versus Vi (n = 4118) versus placebo (n = 4111)	Compared with placebo, all-cause mortality was unaffected by combination therapy (hazard ratio [HR] 0·88; *p* = 0.137) or the components (fluticasone furoate, HR 0·91; *p* = 0.284; vilanterol, 0.96; *p* = 0.655) Rate of decline in FEV1 was reduced by combination therapy 38 mL per year versus 46 mL per year for placebo Combination therapy had no effect on composite cardiovascular events (HR 0.93) No reported excess risks of pneumonia when the groups were compared with placebo
Large observational study [21]	Retrospective cohort study Objective: to assess COPD-related healthcare costs, adherence, and exacerbations in COPD patients	Age ≥ 40 years New initiators of ICS/LABA as either FF/Vi or BUD/FOR for COPD ≥15 months of continuous enrollment	FF/Vi versus BUD/FOR (n = 4513 in each group)	Proportion of days covered (PDC), was significantly better for FF/Vi (0.46) as compared to BUD/FOR: (0.41; *p* < 0.001) indicating better adherence with FF/VI FF/Vi was associated with a 14% lower risk of having a COPD-related moderate or severe exacerbation compared to BUD/FOR (aHR: 0.86, *p* < 0.001) The incidence of exacerbation per 100-person days was 0.33 for FF/Vi 100 compared with 0.40 for BUD/FOR (RR: 0.81, *p* = 0.003)
**Asthma**				
SLS Asthma [22]	Open-label, randomized, controlled, parallel-group effectiveness trial Primary endpoint: % of patients at week 24 with either an ACT score of ≥ 20 or an increase in the ACT score from baseline of ≥ 3 Secondary endpoints: ○ ACT at weeks 12, 24, 40, and 52 ○ Asthma-related primary and secondary care contacts ○ Annual rate of severe exacerbations ○ % of patients who had an increase from baseline of at least 0.5 in total AQLQ score and AQLQ environmental stimuli score, both at week 52	Age: ≥ 18 years Diagnosis of symptomatic Asthma Patients taking regular maintenance therapy with inhaled corticosteroids (ICS) alone or in combination with a long-acting β-agonist (LABA)	FF/Vi (100/25 μg) or FF/Vi (200/25 μg) (n = 2114) versus UC group (n = 2119)	The odds of being a responder were greater in the FF/Vi group versus UC at week 24 (adjusted OR = 2; *p* < 0.0001) The adjusted mean increase from baseline in ACT score was significantly higher with FF/Vi as compared to usual care (adjusted OR = 1.6; *p* < 0.0001) In the primary effectiveness analysis population, the adjusted mean ACT score increased by 4.4 points from in the FF/Vi group compared with an increase by 2.8 points in the usual care group. There were significantly higher responders with respect to AQLQ scores in the FF/Vi group compared to the usual care group at week 52 (OR = 1.79; *p* < 0.0001) No difference in Asthma-related primary and secondary care contacts between the two groups
CAPTAIN [23]	Phase III, randomized, double-blind, active-controlled, parallel-group, study Primary endpoint: change from baseline in clinic trough FEV1 at week 24 Secondary endpoint: Annualized rate of moderate and/or severe Asthma exacerbations over 52 weeks and Asthma Control Questionnaire (ACQ-7) total score. Asthma Respiratory Symptoms were the key secondary endpoints	Age: ≥ 18 years Inadequately controlled Asthma symptoms (ACQ-6 score of ≥1.5) despite maintenance therapy with daily ICS/LABA for at least 12 consecutive weeks Documented health-care contact or documented temporary change in Asthma therapy for acute Asthma symptoms within 1 year Pre-bronchodilator morning FEV1 (30–84%) of predicted normal value and airway reversibility	FF/Vi 100/25 μg group (n = 407) FF/Vi 200/25 μg group (n = 406) FF/UMEC/Vi 100/31·25/25 μg group (n = 405) FF/UMEC/Vi 100/62·5/25 μg group (n = 406) FF/UMEC/Vi 200/31·25/25 μg group (n = 404) FF/UMEC/Vi 200/62.5/25 μg group (n = 408)	Addition of UMEC 62·5 μg to FF/Vi 100/25 μg and FF/Vi 200/25 μg caused least squares mean change from baseline of 110 mL for FF/UMEC/Vi 100/62.5/25 μg and of 92 mL for FF/UMEC/Vi 200/62.5/25 μg in clinic trough FEV1 at week 24 ACQ-7 responder rate was comparable between FF/Vi (100/25) versus FF/Vi (200/25)
Crossover study [13]	Randomized crossover trial Primary Endpoints: Change in FEV1 after 8 weeks Secondary endpoints: Change in other pulmonary function tests, ACQ-5, ACT and FeNO at week 8. The incidence of Asthma exacerbation and adherence barrier questionnaire (Ask-12 survey) were also evaluated after 8 weeks.	Age: ≥ 18 years Patients diagnosed with controlled Asthma as per GINA guidelines Patients who had undergone treatment of two actuations of BUD/FOR DPI combinations (160/4.5 μg) 2 puffs twice daily for at least 3 months	FF/Vi DPI (100/25 μg) 1 puff once-daily versus BUD/FOR DPI treatment (160/4.5 μg) 2 puffs twice-daily	Similar magnitude of change in FEV1 between baseline and week 8 in both groups No significant differences in pulmonary function tests, ACQ-5 scores, ACT scores, and FeNO between baseline and week 8 in both groups Ask-12 score in the FF/Vi DPI group was significantly lower than that in the BUD/FOR DPI group
Summary: The efficacy and safety of FF/Vi in OAD has been established in several large-scale clinical trials including first pragmatic RCT for both Asthma and COPD. FF/Vi in COPD: Reduced rate of exacerbations and rate of decline of FEV1 in Group B/D patients, improved COPD-related health status. FF/Vi in Asthma: Improved Asthma control and quality of life, reduced rescue medication use and better adherence				
